# Man Bites Mosquito: Understanding the Contribution of Human Movement to Vector-Borne Disease Dynamics

**DOI:** 10.1371/journal.pone.0006763

**Published:** 2009-08-26

**Authors:** Ben Adams, Durrell D. Kapan

**Affiliations:** 1 Department of Biology, Kyushu University, Fukuoka, Japan; 2 Center for Conservation and Research Training, Pacific Biosciences Research Center, University of Hawaii at Manoa, Honolulu, Hawaii, United States of America; Yale University, United States of America

## Abstract

In metropolitan areas people travel frequently and extensively but often in highly structured commuting patterns. We investigate the role of this type of human movement in the epidemiology of vector-borne pathogens such as dengue. Analysis is based on a metapopulation model where mobile humans connect static mosquito subpopulations. We find that, due to frequency dependent biting, infection incidence in the human and mosquito populations is almost independent of the duration of contact. If the mosquito population is not uniformly distributed between patches the transmission potential of the pathogen at the metapopulation level, as summarized by the basic reproductive number, is determined by the size of the largest subpopulation and reduced by stronger connectivity. Global extinction of the pathogen is less likely when increased human movement enhances the rescue effect but, in contrast to classical theory, it is not minimized at an intermediate level of connectivity. We conclude that hubs and reservoirs of infection can be places people visit frequently but briefly and the relative importance of human and mosquito populations in maintaining the pathogen depends on the distribution of the mosquito population and the variability in human travel patterns. These results offer an insight in to the paradoxical observation of resurgent urban vector-borne disease despite increased investment in vector control and suggest that successful public health intervention may require a dual approach. Prospective studies can be used to identify areas with large mosquito populations that are also visited by a large fraction of the human population. Retrospective studies can be used to map recent movements of infected people, pinpointing the mosquito subpopulation from which they acquired the infection and others to which they may have transmitted it.

## Introduction

Our understanding of diseases such as malaria, yellow fever, onchocerciasis and filiarisis was profoundly affected when the medical scientists of the late 1800s revealed the role of insects as intermediate hosts known as vectors – carriers of disease from one primary host to another [1]. Subsequently, researchers interested in controlling these and other vector-borne diseases have rightly focused on the insect vector as the critical link between infected and susceptible hosts. A careful combination of insect population biology and mathematical modeling has produced a number of important advancements. Early mathematical analysis of malaria transmission, for instance, revealed disease incidence to be most sensitive to survival of adult female mosquitoes and led to DDT based intervention strategies that eradicated malaria from large parts of the world [2].

Nevertheless, vector-borne diseases remain a significant problem, even in highly modernized industrial cities. Singapore, for example, has for many years implemented a vigorous program of domestic vector source reduction and insecticide spraying in a full GIS-enabled public health protection effort. Nevertheless dengue continues to circulate and, after a brief period of respite, outbreaks are becoming increasingly severe [3]. In this article we aim to offer additional insight into the modern urban epidemiology of vector-borne disease. We shift focus from the vector to the host and develop a mathematical model to investigate the impact of human movement and mosquito patchiness on the dynamics and persistence of vector-borne disease at the city scale. Key issues for disease control in cities where successful vector control strategies have led to an overall rarity of mosquitoes include identifying reservoirs for the virus and understanding how it circulates in the urban environment. Urban mosquito populations may be patchily distributed [4] particularly in the presence of control activity. Data from Puerto Rico show that human cases are clustered at the scale of households, where domestic mosquitoes are responsible for transmission, but not at the scale of city blocks [5]. So, beyond individual households, persistent sources of infection and the routes by which it is spread remain unclear.

Given that mosquitoes are responsible for dengue transmission, mosquito movement may have a role in connecting such patches to create reservoirs of the virus and disseminate it widely through the human population. However, the primary urban insect vector is *Aedes (Stegomyia) aegypti (L.)*. This species is highly anthropophilic and, while capable of relatively long flights if hosts or oviposition sites are unavailable locally [6], rarely travels more than a few tens of meters throughout its lifetime [7], [8], [9] but see [10]. In comparison with mosquitoes, people inhabiting urban and semi-urban environments move frequently and over large spatial scales. The dispersal of directly transmitted infections, such as measles or influenza, is clearly due to human movement at all spatial scales. The role of humans as medium to long distance carriers of dengue has been documented many times [11]. It is therefore highly plausible that people play a key role in the spatial spread of dengue in urban areas, carrying the infection between patchily distributed mosquito communities. As a result, community transmission may be disconnected from local transmission identified by case clusters based on home address. Instead, the infection may be passed via the mosquito population between any two people that visit the same place, even if they are never there at the same time [12]. Here we investigate this hypothesis using a metapopulation model.

Metapopulations are groups of interconnected populations that are subject to semi-independent local dynamics. The metapopulation concept has been used extensively in conservation biology, ecology, epidemiology, evolution and population genetics. It has improved our understanding of a myriad of phenomena including population persistence, genetic drift, local adaptation and speciation [13], [14], [15]. The fundamental concept is the rescue effect. Locally, subpopulations frequently become extinct. However, asynchronous dynamics in multiple subpopulations mean that barren patches are regularly reseeded and the probability of global extinction is reduced.

Metapopulation theory has been successfully applied to epidemiological problems involving directly transmitted diseases [15], [16], [17]. The frequency of measles in English towns and cities has been shown to depend on the local population size. Large cities support endemic circulation and periodically seed epidemics in smaller towns [18]. Similarly, the states of the USA can be considered as subpopulations linked by commuter movement. This framework has been used to show that influenza epidemics in populous states lead to widespread, synchronized epidemics throughout the metapopulation. Epidemics in sparsely populated states only lead to sporadic outbreaks elsewhere [19]. More general studies have shown that disease persistence is maximized when connectivity between patches is of intermediate intensity as there must be a balance between the asynchrony of subpopulation dynamics and the frequency of seeding events [15], [16], [17].

For vector-borne diseases, metapopulation models have been applied to situations in which humans form static subpopulations connected by mobile zoonotic host or vector populations. Studies indicate that large outbreaks of bubonic plague are more likely where rat movement results in very weakly connected human subpopulations [20], [21]. With malaria, frequency dependent biting has been shown to enhance persistence in situations where several patches of non-mobile hosts are connected by a well-mixed mosquito population [22]. A study based on a simple model of two static vector populations connected by mobile humans concluded that even low transmission areas are prone to dengue epidemics if local residents also visit high risk areas [23].

To develop our understanding of the impact of human host movement on mosquito borne disease dynamics we construct and analyze a series of metapopulation models. In these models the human population is assumed to live in a home patch free of mosquitoes but moves to and fro patches with immobile mosquito subpopulations. There is no explicit distance or spatial arrangement of the patches, but human movement connects them. This framework is intended as an abstraction of the network structure arising from commuting patterns in an urban environment. We use it to show that local mosquito populations can act as short-term reservoirs of infection and people act as efficient carriers of infection between these reservoirs. Therefore, community transmission can lead to efficient endemic circulation even in the absence of local, home based, transmission.

## Methods

### Mathematical model

**Figure 1:**
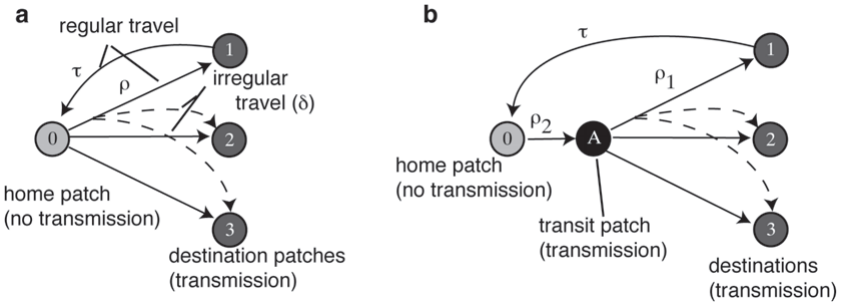
Network for models with 3 destination patches. a: basic model, people travel directly from home patch to destination and back. b: transit patch model, all people pass through the same transit patch (A) en route to their destination. Solid lines indicate regular travel patterns to (rate *ρ*) and fro (rate *τ*) patch 0 and patch *j*. For clarity the return route is only shown for patch 1. Dashed lines indicate irregular travel patterns, the frequency of which is controlled by *δ*. For clarity these have been omitted for the subpopulations regularly travelling to patches 2 and 3.

**Figure 2:**
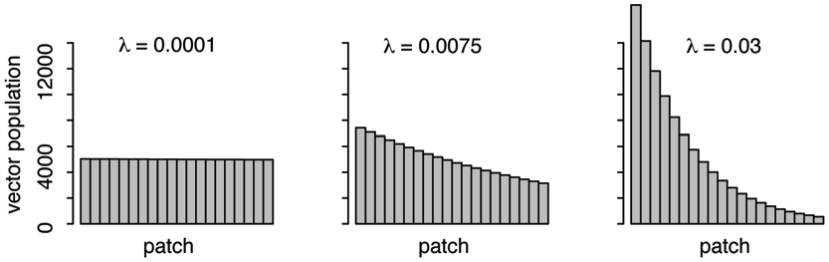
Role of parameter *λ* in determining the distribution of total mosquito population *N^v^* = 100,000 between *n* = 20 patches (Supplementary Information S1 equation 2). Values of *λ* close to 0 give an almost uniform distribution, larger values of *λ* give increasingly skewed distributions.

Here we outline the key elements of the mathematical model used in this study. A detailed mathematical description is given in the Supplementary Information S1. The meanings of all variables and parameters are summarized in Table 1. We extend established metapopulation models for directly transmitted infections [16], [24], by considering a basic structure composed of *n*+1 patches labeled 0, 1, …*n* as shown in Figure 1a. The widespread use of modern mass transportation systems mean that, at the scale of a city, distance is a weak indicator of human movement patterns. So there is no spatial relationship between patches. To highlight the role of community transmission domestic mosquito control and prophylactic measures are assumed to be efficient and prevent all domestic (home) transmission. Patch 0 is designated the ‘home’ patch for the entire human population. Only one such patch is required because the absence of local transmission dynamics renders any spatial substructure irrelevant to the objectives of this study. A mosquito population is associated with each of the remaining *n* ‘destination’ patches. There is no mosquito migration between these patches because there is no assumption of spatial proximity. Furthermore, as detailed in the Introduction, relative to humans *Ae. aegypti* dispersal is poor. The mosquito population is divided into *n* subpopulations *N^v^_i_* and has a total size of *N^v^*. To model changes in relative population size the evenness of this division is controlled by the parameter *λ*. As shown in Figure 2, for *λ* = 0.0001 the distribution is almost uniform, for *λ* = 0.0075 it is almost linear and for *λ* = 0.03 it is highly skewed.

**Table 1:** Parameter values used throughout this paper.

Symbol	Meaning	Normal value	Range
*N^h^*	Total human population	100,000	-
*μ_h_*	Human death rate	0.0000457	-
*ε_h_*	Incubation rate in human	0.2	0–∞
*γ*	Human recovery rate	0.2	-
*N^v^*	Total mosquito population	50,000	50,000–250,000
*μ_v_*	Mosquito death rate	0.143	-
*ε_v_*	Incubation rate in mosquito	0.143	-
*β*	Mosquito biting rate	0.33	-
*n*	Number active patches in addition to home patch	50	1–50
*ρ*	Transfer rate patch 0 to patch *j*	1	-
*τ*	Transfer rate patch *j* to patch 0	1	0–10
*δ*	Degree of human mixing between patches	0	0–1
*λ*	Skew of mosquito population distribution	0.0001	0.0001–0.03

Where no range is given this parameter always takes the same value. All rates are per day.

The total human population *N^h^* is fixed at 100,000. It is divided into *n* subpopulations *N^h^_j_* where *j* = 1…*n* is the usual destination patch of that group. Each subpopulation is subdivided into a further *n*+1 subpopulations *N^h^_ij_* where *i* = 0…*n* is the current patch of those individuals. People in patch 0 leave at rate *ρ* and travel to their usual destination patch with probability (1−*δ* )+*δ/n*. They travel to one of the other *n*–1 patches each with probability *δ/n*. Thus *δ* reflects the degree to which variation in human movement patterns, or mixing, connects otherwise distinct mosquito populations. People in patch *i* ≠ 0 leave at rate *τ* and return directly to patch 0. Thus *ρ* and *τ* determine the proportion of time spent in each patch. Throughout this paper *ρ* is fixed to be 1. So, on average, the entire population leaves the home patch each day. Then *τ* controls the length of time spent in the destination patches. For simplicity this is taken to be the same for all patches. Larger values of τ correspond to shorter times spent away from home. Larger values of τ lead to a larger instantaneous population size in patch 0 and smaller instantaneous population sizes in the other patches.

A standard host-vector type model for disease transmission is integrated into this metapopulation structure [25]. Each host subpopulation is subdivided according to infection status: susceptible (*S^h^_ij_*), exposed (infected but not infectious, *E^h^_ij_*), infectious (*I^h^_ij_*) and recovered (*R^h^_ij_*). Hosts of all classes die at constant rate *μ_h_* and are replaced with susceptible hosts. The total size of each subpopulation remains constant. Infected hosts become infectious at rate *ε_h_*. Infectious hosts recover at rate *γ*. Recovered hosts have complete lifelong immunity to re-infection. All hosts continue to move at the same rate regardless of their infection status. This approximation is reasonable given high levels of mild or asymptomatic infection as has been observed, for example, with dengue ([26] and see Discussion). Each vector subpopulation is subdivided into susceptible (*S^v^_i_*), exposed (*E^v^_i_*) and infectious (*I^v^_i_*) classes. Vectors of all classes die at constant rate *μ_v_* and are replaced with susceptible vectors. The size of the subpopulation remains constant. Exposed vectors become infectious at rate *ε_v_* and remain in this class until they die. Transmission may occur when a vector bites a host. The biting rate per vector is given by *β*. Since *Ae. aegypti* almost exclusively bites humans the total number of bites at the population level is not likely to be limited or controlled by the availability of hosts. So transmission is frequency, rather than density, dependent, as discussed in detail elsewhere [27], [28], [29], [30]. This means the local vector-host transmission rate is determined by the absolute number of infectious vectors in that patch and the proportion of the visiting host population that is susceptible. The local host-vector transmission rate is determined by the absolute number of susceptible vectors in that patch and the proportion of the visiting host population that is infectious.

## Results

We begin by considering the simplest possible model composed of the home patch (0) and a single destination patch (1). We then expand our analysis to consider networks with 3 and 50 destination patches. We also consider the impact of ‘transit’ patches that could serve as hubs of infection. We conclude with stochastic simulations to understand how patch dynamics affect pathogen persistence time in a system of 50 patches.

### One destination patch

#### The impact of host residency times and vector populations sizes on pathogen transmission

**Figure 3:**
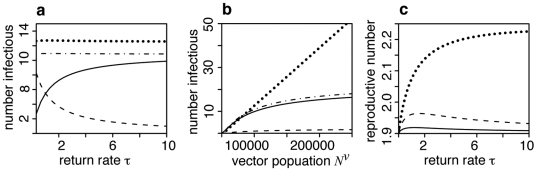
Endemic equilibrium solutions and basic reproductive number of model with one destination patch. a, b: Number of infectious hosts in patch 0 (*I^h^*_0_, solid line), patch 1 (*I^h^*_1_, dashed line) and in total (*I^h^*_0_+*I^h^*_1_, dot-dash line) and the number of infectious mosquitoes in patch 1(*I^v^*, dotted line) as functions of the rate at which hosts leave patch 1 (*τ*) and mosquito population size (*N^v^*). Larger values of *τ* correspond to a shorter residence time in patch 1. c: Basic reproductive number (*R*_0_^2^) as a function of *τ*. Line styles indicate the within-host incubation rate: *ε_h_* = 0.2 (solid), 1 (dashed), 200 (dotted). The duration of incubation is 1*/ε_h_* days.

With a single destination patch host mixing between patches, parameterized by *δ*, and the evenness of the mosquito distribution, parameterized by *λ*, are not applicable. Figure 3a shows how the steady state numbers of infectious hosts and vectors depend on*τ*, the rate of return to patch 0. Recall that larger values of *τ* correspond to a shorter residence time in patch 1. As *τ* increases the number of infectious hosts increases in patch 0 and decreases in patch 1. This is a consequence of the movement regime. The total number of infectious hosts remains constant, as does the number of infectious vectors. So, although new infections can only occur in patch 1, the total number of infections is independent of the time hosts spend there.

Figure 3b shows that as the total number of vectors *N* ^v^ increases the number of infectious hosts begins to saturate but the number of infectious vectors increases linearly. This difference is also due to frequency dependent biting. The number of susceptible vectors that become infected by biting infectious hosts is linearly related to the size of the susceptible vector population. Since infection levels are low, there is no immunity, and demographic turnover is rapid, almost all of the vector population is susceptible. The number of infectious vectors that bite susceptible hosts is also linearly related to the vector population size. In this case, as infection incidence increases more hosts become immune. So most bites do not actually result in transmission.

The basic reproductive number *R*_0_ is defined as the expected number of secondary infections resulting directly from a single infected individual in an otherwise naïve population [25]. Formally, in host-vector models it does not matter whether these infections occur in a host or a vector [31]. However, it is conventional to modify the definition such that *R*_0_^h^ becomes the host reproductive number: the expected number of secondary host infections resulting from a single infected host, with the intermediate vector infections remaining implicit. This has also been formalized as the type reproductive number *T*_1_ [31], [32]. A similar definition provides the vector reproductive number *R*_0_^v^.

In a standard single patch host-vector model *R*_0_^h^ = *R*_0_^v^ = *R*_0_^2^ and it does not matter if the initial infected individual is a host or a vector. However, in the two-patch model considered here, the location of an initial infected host is important. Suppose, on average, an infected vector infects a total of *h*_1_ hosts. Suppose also a host infected in patch 1 infects a total of *v*_1_ vectors. Then, one initial infected vector in patch 1 will infect *h*_1_ hosts. All of these people are in patch 1 and go on to infect *v*_1_ vectors. This process leads to a total of *h*_1_ *v*_1_ new vector infections. Similarly, one initial infected host in patch 1 will infect *v*_1_ vectors. These will infect *h*_1_ hosts, leading to a total of *h*_1_ *v*_1_ new host infections. Hence *R*_0_^h^ = *R*_0_^v^. If, however, the initial infected host is in patch 0, it is expected to spend slightly less of the infectious time in patch 1 due to a waiting period before traveling there. Hence, this host will infect less than *v*_1_ vectors, leading to fewer than *h*_1_ *v*_1_ new host infections. Therefore *R*_0_^h^ will be slightly smaller than *R* **_0_^v^**. A technique for constructing a global *R*_0_ known as the next generation method overcomes this problem by moving past the transitory dynamics of the initial introduction and, in a sense, averaging over many subsequent generations [33], [34]. Similar methods are applied when considering the spread of diseases in social contact networks [35].

The global basic reproductive number of the model with one destination patch (see Supplementary Information S1) depends in a complex way on *τ* and *ε_h_* as shown in Figure 3c. When there is no incubation in the host (*ε_h_* → ∞) *R_0_* increases monotonically as *τ* increases. However, when the incubation period is similar to the patch residence time, the basic reproductive number peaks when *τ* is around 1 and decreases slightly thereafter. A delay between infection and infectiousness means that the host may leave patch 1 and not return until some time after becoming infectious. This reduces its transmission potential. The implication of this unimodality in the reproductive number is that the mosquito population required for the disease to be endemic is smallest when people spend an intermediate amount of time in the transmission patch.

### Three destination patches

We now expand the model to three destination patches, allowing host mixing between patches (*δ*) and variation in the vector population distribution (*λ*). With the addition of multiple patches the complexity of the mathematical system increases dramatically. To simplify we turn to an approximate form derived using a method originally suggested for an epidemiological metapopulation model with direct transmission [16]. This approach is based on the observation that the timescale of human travel is much faster than the timescale of the epidemiological dynamics. Therefore we can approximate the size of each host subpopulation (*S_ij_*, *E_ij,_ I_ij,_ R_ij_*) in each patch by assuming that it scales proportionally with the total population expected to be in that patch at equilibrium. Thus we define *S^h^_j_* to be the total number of susceptible hosts with normal destination patch *j*, irrespective of their current location. Similar definitions apply for *E^h^_j_*, *I^h^_j_*, *R^h^_j_* and *N^h^_j_*. The variables for the vector populations in patch *j* (*S^v^_j_*, *E^v^_j_*, *I^v^_j_*) are unchanged. Note that the movement rate parameters *ρ* and *τ* drop out of the approximate system entirely. Complete equations are given in the Supplementary Information S1.

Here we focus on the potential for pathogen transmission in the system as summarized by the basic reproductive numbers. Locally, as before, the expected number of secondary host infections resulting from a single infected host (i.e. host-vector-host transmission) will not necessarily be the same as the expected number of secondary vector infections resulting from a single infected vector (vector-host-vector transmission). Furthermore, with multiple transmission patches, the normal destination of the initial infected host, or the residence patch of the initial infected vector may also be important. The next generation method can still be used to calculate the global reproductive number *R*_0_. It is also instructive to derive the reproductive numbers associated with the initial phases of an epidemic due to the introduction of a single infectious host or vector. We now define the host reproductive number *R*_0_^h^_j_ as the total number of host infections resulting from a single infected host with normal destination *j*. The vector reproductive number *R*_0_^v^_j_ is defined similarly.

### The impact of vector distributions and host mixing on pathogen transmission

**Figure 4:**
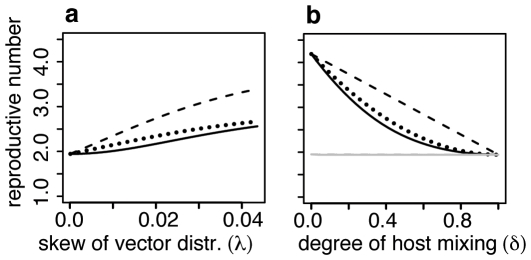
Reproductive numbers of the approximate model with 3 destination patches evaluated in the patch with largest vector population. a: as a function of the degree of skew in the vector population distribution (λ) when host mixing is intermediate (*δ* = 0.5). b: as a function of the degree of host mixing (*δ*) when the vector population distribution is highly skewed (*λ* = 0.03, black lines) and uniform (*λ* = 0.0001, overlapping grey lines). Solid line is the global *R*_0_^2^ for the entire metapopulation calculated using the next generation method. The dashed line is the host reproductive number (*R*_0_^h^_j_) associated with the patch (*j*) with the highest mosquito density. The dotted line is the vector reproductive number for the same patch (*R*_0_^v^_j_).

The global reproductive number, along with the host and vector reproductive numbers for the patch with the largest vector population are shown as functions of the skew in the vector distribution (*λ*) in Figure 4a and degree of host mixing (*δ*) in Figure 4b. When the vector population is uniformly distributed the global, host and vector reproductive numbers all agree. Furthermore, the degree of host mixing has no influence because all patches have identical transmission potential. Increasing the skew of the vector distribution increases all reproductive numbers. In the patch with the largest mosquito population the host reproductive number *R*_0_^h^_j_ is much bigger than that of the vector *R*_0_^v^_j_. Both are larger than the global reproductive number for the metapopulation. This indicates that in patches with large mosquito populations, an infected person has greater influence on the maintenance of the pathogen than an infected mosquito.

In patches with small mosquito populations the situation is reversed. The vector reproductive number is larger than the host reproductive number. Both are smaller than the global reproductive number (not shown). Host mixing has no impact when the vector distribution is uniform. For skewed vector distributions increased host mixing reduces all reproductive numbers. The global *R*_0_ is similar to the vector reproductive number in the patch with the largest subpopulation. Both are smaller than the host reproductive number, which decreases linearly as host mixing increases. When there is no host mixing (*δ* = 0) or complete host mixing (*δ* = 1) all reproductive numbers converge. This relationship indicates that, in patches with large mosquito populations, the difference in the influence of infected people and infected mosquitoes for the maintenance of the pathogen is greatest when the extent to which people mix among patches is intermediate.

### Relative importance of transmission within and between patches

**Figure 5:**
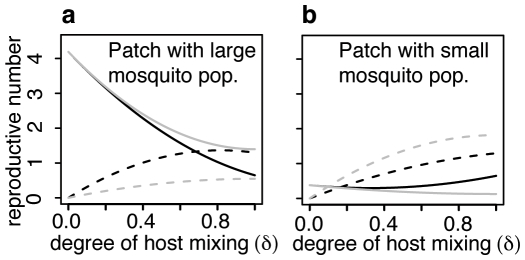
Components of the host and vector reproductive numbers (*R*_0_^h^_j_, *R*_0_^v^_j_) of the approximate model with three destination patches and a highly skewed (*λ* = 0.03) vector population evaluated in the patches with the largest (a) and smallest (b) vector populations as a function of the degree of host mixing (*δ*). The initial infected individual is in patch *j*. Solid line is the component of the reproductive number related to transmission within patch *j*. Dashed line is the component related to transmission to patches other than *j*. Black lines correspond to the host-vector-host transmission cycle. Grey lines correspond to the vector-host-vector transmission cycle.

Further insight comes from considering the composition of the reproductive numbers in terms of transmission within the patch i.e. secondary infections occurring within the same subpopulation as the primary infection, and dissemination between patches i.e. secondary infections occurring in different subpopulations to the primary infection. The mathematical details are set out in the Supplementary Information S1. Figure 5 shows how the within and between patch components of the host and vector reproductive numbers depend on the degree of host mixing (*δ*) if the vector population distribution is highly skewed. When there is no host mixing transmission can only occur within the patch where infection originates. As host mixing increases, transmission becomes less likely in the patch were infection originates and more likely in other patches. For patches with large vector populations, if mixing is strong the majority of secondary host infections (i.e. host-vector-host infections) occur in hosts associated with other patches. However, the majority of secondary vector infections (i.e. vector-host-vector infections) are always in the same patch, regardless of host mixing. In patches with small vector populations, the local transmission cycles are less influential. In both host and vector populations, the majority of secondary infections occur outside of the patch where the infection originated unless host mixing is very weak.

To understand the relative importance of transmission within and between subpopulations consider the transmission process by which an infected vector in patch *j* maintains the disease in the local subpopulation. Note that the efficiency of this process increases as vector subpopulation size in patch *j* increases, but is not related to the size of the entire vector metapopulation (Supplementary Information S1 equation S16). Now consider the process by which an infected host associated with patch *j* maintains the disease within the local subpopulation. Note that the efficiency of this process increases when either the vector subpopulation in patch *j* or the entire vector metapopulation increase (Supplementary Information S1 equation S14). The difference arises from the way in which infections are spread to subpopulations not associated with patch *j* and then back again. To see this, start with an infected vector in patch *j*. Frequency dependent biting means the number of hosts associated with patches other than *j* that are subsequently infected while visiting patch *j* is independent of any vector subpopulation size. The number of vectors in patch *j* then infected by those hosts is directly proportional to the total number of vectors in patch *j*. Now start with an infected host associated with patch *j*. The number of vectors subsequently infected by this host in patches other than *j* is directly proportional to the number of vectors in those patches. The distribution of the vector population outside of patch *j* is not important. This is because a host associated with patch *j* travels to any patch other than *j* with equal probability. Therefore it is equally likely to encounter any individual vector. The number of type *j* hosts these vectors then infect is independent of any vector population size.

The efficiency with which an infected vector in patch *j* spreads the infection to other vector subpopulations decreases if the size of the vector population in patch *j* increases. But it increases if the size of the entire vector metapopulation increases (Supplementary Information S1 equation S18). In contrast, the efficiency with which an infected host associated with patch *j* spreads the infection to other host subpopulations increases if either the vector population in patch *j* or the entire vector metapopulation increase (Supplementary Information S1 equation S15). To understand this, consider an infected vector in patch *j*. The number of hosts, associated with any patch, that are subsequently infected is independent of any vector population size. This is because hosts are equally likely to travel to any of their irregular destination patches. Hence the number of vectors in patches other than *j* then infected by those hosts is directly proportional to the size of the entire metapopulation excluding patch *j*. So concentrating a greater proportion of the vector population into patch *j* reduces the potential transmission to other patches. Increasing the total metapopulation size has the opposite effect because it leads to a proportional increase in the number of vectors in each patch. Now, start with an infected host associated within patch *j*. The number of vectors subsequently infected in patch *j*, and in the wider metapopulation, is directly proportional to the number of vectors in those populations. The number of hosts of type other than *j* these vectors then infect is independent of any vector population size. The distribution of the vector population outside of patch *j* is not important because a host associated with patch *j* travels to any patch other than *j* with equal probability.

### Fifty destination patches: the rescue effect and pathogen persistence

**Figure 6:**
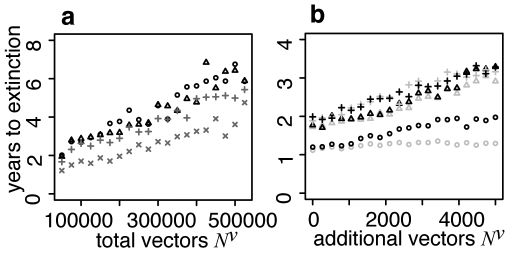
Mean time to extinction, in years, calculated by applying a stochastic solver to the approximate version of the model with discrete host and vector populations and 50 patches. Each plotted point is the average of 100 trials. The initial conditions were constructed by applying a deterministic solver to find the endemic equilibrium and rounding up all fractional population sizes. Where the endemic equilibrium was unstable, the disease free equilibrium was modified so that there was one infected and infectious host and vector in each patch. a: Time to extinction as a function of the total vector population *N^v^* in the basic model. Pluses: skewed vector distribution (*λ* = 0.03) and weak host mixing (*δ* = 0.1). Crosses: uniform vector distribution (*λ* = 0.0001) and weak host mixing. Triangles: skewed vector distribution and strong host mixing (*δ* = 0.9). Circles: uniform vector distribution and strong host mixing. b: Time to extinction as a function of the number of vectors additional to 50,000 in the total vector population for the model with a transit patch. Black – additional vectors all in transit patch (A). Grey – additional vectors evenly divided between normal destination patches (control). Circles – no host mixing between destination patches (*δ* = 0). Triangles – weak host mixing (*δ* = 0.1). Crosses – strong host mixing (*δ* = 0.9). Except for transit patch, vector population evenly distributed between 50 destination patches.

The deterministic models studied so far have offered some insight into how human movement connecting mosquito subpopulations affects the expected occurrence and prevalence of disease. In order to examine disease persistence we also implemented a stochastic version of the approximate model with fifty destination patches and discrete variables for each population group. From an initial condition close to the deterministic equilibrium the model was iterated using a continuous time Markov process [36]. Each simulation was continued until the *E^h^*, *I^h^*, *E^v^*, and *I^v^* subpopulations in all patches were zero and the system was disease free. Figure 6a shows how the average number of years until disease extinction depends on the total vector population size. Extinction is most rapid when host mixing is weak and the vector distribution is skewed. It takes longest when host mixing is strong, regardless of the vector population distribution. In all cases, increasing the total vector population size leads to an approximately linear increase in the time to extinction. Figure 6 only shows results for very weak and very strong mixing but we found that the time to extinction is always greater for larger values of *δ*. We did not find it to be optimized at an intermediate value.

**Figure 7:**
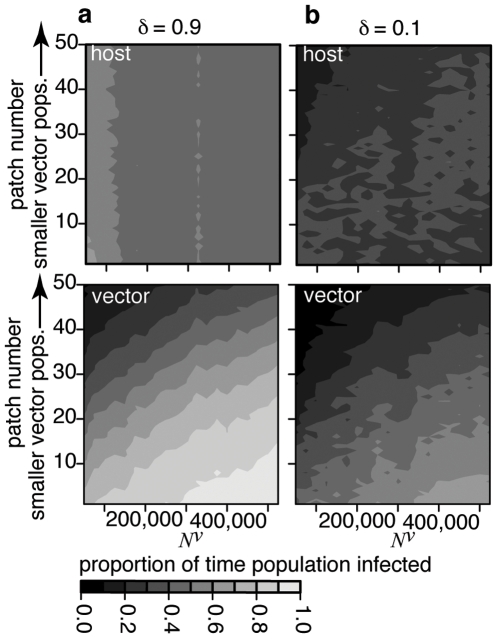
Patch occupancy expressed as the mean proportion of the total time to extinction that there is at least one infection in the host (*E^h^* or *I^h^*) or vector (*E^v^* or *I^v^*) in each patch as a function of the total vector population *N^v^*. Calculated using a stochastic solver for the approximate version of the model with 50 patches, a: strong (*δ* = 0.9) and b: weak (*δ* = 0.1) host mixing and a skewed (*λ* = 0.03) vector distribution between patches. Paler shades indicate a subpopulation is infected for a greater proportion of time. Each point is the average of 25 trials with initial condition close to the endemic equilibrium found using a deterministic solver.

In order to gain further insight, we defined host patch occupancy as the proportion of the total time between disease introduction and extinction that there is at least one infected or infectious host in the patch. Vector patch occupancy was defined similarly. If the vector population is uniformly distributed, whether host mixing is strong or weak, increasing the size of the vector metapopulation leads to a large increase in vector patch occupancy. There is little change in host patch occupancy (not shown). The difference arises because the accumulation of immunity in the host population maintains approximately constant infection incidence. In the vector population, the absence of immunity and rapid demographic turnover mean infection incidence is proportional to population size. Patch occupancy when the vector distribution is skewed is shown in Figure 7. When mixing is strong, host patch occupancy is almost uniform. It is, at best, very weakly related to either vector population size or vector patch occupancy. In contrast, vector patch occupancy is generally higher. It increases considerably when either the local vector population, or the global vector population, size increases. Again the difference is due to the presence or absence of immunity. When mixing is weak, the general trend is the same although less striking. Host and vector patch occupancy are also lower.

### Alternative network structure: transit patch

The main body of our analysis has concerned a network structure in which people travel between a home patch where mosquitoes are absent and other patches where mosquitoes are present. People that vary their travel patterns transmit infection between mosquito populations. There are many other possible network structures, but here we consider just one simple alternative. As in the previous model hosts move between a home patch where vectors are absent and other patches where vectors are present. Regardless of their final destination, all hosts must pass through the same transit patch (A), where vectors may be present. For simplicity we assume that the transit patch is only used on the outward part of the journey. Thus hosts travel from patch 0 to patch A at rate *ρ*_1_, from patch A to their destination patch at rate *ρ*_2_ and then return to the home patch directly at rate *τ*.

Our main interest here is the system where hosts always travel to the same destination. In this case there is no mixing between patches but the transit patch vector population *N^v^_A_* acts as a hub and a reservoir of infection. We will also consider how this mode of transmission between vector subpopulations interacts with transmission by hosts varying their destination patch, as before represented by *δ*. A diagram of this network is shown in Figure 1b. In order to reduce the system to a manageable number of equations we constructed approximate forms as before. This approximation eliminates *ρ*_1_, *ρ*_2_, and *τ*, as detailed in the Supplementary Information S1.

We assume that the total vector population excluding the transit patch subpopulation is uniformly distributed between the *n* destination patches. So each patch contains *N^v^/n* vectors. We also assume hosts always travel to the same destination patch, so *δ* = 0. Then the global reproductive number of the system can be found using the next generation method (see Supplementary Information S1). The transit patch vector subpopulation appears additively in the reproductive number. This structure means that each individual vector in the transit patch has the same importance as an individual in any other patch. So, even if the vector populations in the places where people spend most of their time are reduced below the local threshold for infection to be endemic, a large vector population in an area that all hosts pass through regularly may be sufficient to ensure continued transmission.

The stochastic form of the model was used to assess the impact that vectors in the transit patch have on disease persistence. The total vector population size in all destination patches was set at 50000. This number is slightly below that required for global *R*_0_>1. Additional vectors were then added, either together as a single subpopulation in the transit patch, or distributed evenly between all destination patches. The second of these experiments acts as a control because the transit patch remains empty but the total vector population still increases.

Figure 6b shows the extinction times associated with adding between 0 and 5000 vectors. When there is no host mixing the transit patch has a striking impact on disease persistence. The control experiment shows that, when there is no transmission in the transit patch, the time to extinction remains roughly constant even when the total vector population in the destination patches is increased by 5000. In contrast to this, the time to extinction nearly doubles when 5000 vectors are added to the transit patch. The mixing of people in the transit patch means that the mosquito subpopulation there is constantly re-infected. It then acts as a source for the repeated reseeding of brief outbreaks in the destination patches. The impact of the transit patch is very clear when hosts always travel to the same destination patch and so cannot spread the infection directly between vector subpopulations. However, even a small amount of variation in travel destination (*δ* = 0.1) overrides the transit patch effect. In this case, the time to extinction increases by a similar amount as the number of vectors increases, whether these additional vectors are in the transit patch or scattered through the destination patches.

## Discussion

Over the last century human population growth and urbanization has been accompanied by a huge increase in mobility. The combined effect of these factors means that, despite great improvements in hygiene, sanitation and vector control, containment of disease remains one of the biggest challenges of the modern world. The importance of increased human mobility cannot be underestimated. On national to continental scales the airline transport network has played a key role in the global dissemination of influenza and SARS [37]. Migrants, tourists and commercial travelers have a big influence on the spread of HIV [38]. On a more local scale, human movement in metropolitan areas is frequent and extensive but often composed of highly structured commuting patterns between homes and places of employment, education or commerce. In this article we have examined how this type of movement can affect the occurrence and persistence of vector-borne pathogens. We suggest that this is a vital factor to consider in the ongoing development of strategies to eradicate vector-borne diseases such as dengue from urban centers [39].

In the simplest metapopulation we considered, all people commute between a mosquito-free home patch and a single destination patch with a resident mosquito population. Analysis of this model showed that infection prevalence in both the human and mosquito populations is almost independent of the time people spend in the transmission patch. This may be because the biting rate is frequency dependent and, regardless of the density of the human population, a mosquito will bite the same number of people per day. It follows that cursory inspection of data detailing human and mosquito population sizes may not reveal centers of disease transmission. A patch with short residence times may appear to be occupied by few people and harbor even fewer infected people. However, over the course of the day, a large part of the wider community may pass through the patch and infectious people may transmit to the local mosquito population. Since the mosquitoes do not move, the infection may remain long after the people have departed. This persistence in the mosquito population can make such patches transmission centers even though almost of the resultant human infections will actually be sampled in the home patch, where there is no transmission.

When the mosquito population is divided into multiple distinct subpopulations, skewed distributions increase the transmission potential of the pathogen, quantified in the basic reproductive number. The largest subpopulation has an over dominant impact on the transmission potential throughout the entire metapopulation. The distribution of the mosquito population also modifies the impact of the human-mediated connectivity between subpopulations. When the distribution is uniform the infection probabilities are the same wherever a person goes. The expected endemic incidence is independent of the degree of connectivity. When the distribution is skewed, higher connectivity leads to lower endemic incidence in patches with large mosquito subpopulations but higher incidence in patches with small subpopulations. If connectivity is weak, people that travel regularly to patches with large mosquito populations form a high risk group. They have a relatively high level of infection compared to people that travel regularly to patches with small mosquito populations. As people vary their travel patterns more frequently connectivity strengthens. People become more loosely associated with a particular patch. Infection incidence falls in the high risk group because they spend more time in patches with smaller mosquito populations. Incidence grows in the low risk group. The net effect is a reduction in the overall incidence.

More extensive variation in human movement patterns causes the degree of connectivity between mosquito subpopulations to increase. It moderates the dominant effect the largest mosquito subpopulations have on transmission. More variable human movement also enhances pathogen persistence. It extends the duration of endemic circulation in the metapopulation as a whole. Due to frequency dependent biting, larger mosquito populations accommodate more infected mosquitoes and so function as better disease reservoirs. More variable human movement makes it more likely that people will carry the infection from these areas to mosquito subpopulations where the pathogen has died out.

Classical theory indicates that intermediate connectivity should optimize persistence. Very weak connectivity should compromise persistence because ‘rescues’ are less likely. Very strong connectivity should compromise persistence because the dynamics of subpopulations become synchronized. In contrast, we found that persistence in our host-vector metapopulations is always enhanced by greater connectivity. It is possible that this is because any synchronization in the epidemiological dynamics that would lead to simultaneous low incidence in mosquito subpopulations is disrupted by the high rate of demographic turnover in these populations. This rapid turnover is also evident in the proportion of time for which each subpopulation harbors the pathogen. Despite containing the same number of individuals, mosquito subpopulations are much more likely to contain infection than the associated human subpopulations. Almost all mosquitoes are susceptible because mosquito turnover occurs on the same timescale as the infection dynamics. Conversely, a large proportion of people are immune, effectively inert, because host demographic turnover is so slow.

We also adjusted the network structure so that all people pass through the same transit patch en route to their final destinations. Analysis of the basic reproductive number indicated that the mosquito population in such a patch can play a critical role. When all destinations support similar mosquito subpopulations and people always travel to and fro the same destination, a mosquito in the transit patch makes the same contribution to basic reproductive number as a mosquito elsewhere in the metapopulation. A large mosquito population in a frequently visited area may be sufficient to ensure infection is endemic, even if there are relatively few mosquitoes elsewhere. When people do not vary their travel patterns and there is no direct connectivity between subpopulations the transit patch can significantly enhance disease persistence in the metapopulation by acting as a reservoir and hub. If people vary the patch they visit even occasionally, the effect of the transit patch is overridden.

The model framework we have introduced here employs several assumptions that we believe can be relaxed without qualitatively changing our conclusions. We have assumed that efficient vector control means there is no transmission in the home patch and all people are equally likely to travel from the home patch to the destination patch. Allowing low-level transmission in the home patch would cause it to act in a similar way to a transit patch. We have shown a transit patch only has a significant impact when there is no variation at all in human travel patterns. Partitioning the population so that some people always stay at home would enhance the potential for the home patch to act as a hub and reservoir. However, the mosquito population would need to be large for home transmission to dominate over transmission in other areas with large mosquito populations. Empirically, a quick assessment of the relative importance of ‘home’ versus ‘community’ transmission is possible using established methods based on the measurement of transmission chain length of case ‘clusters’ centered on home address [40].

We also assumed that mosquitoes will bite people whenever they are in the same location. Empirical studies in Trinidad found that the *Ae. aegypti* biting frequency is trimodal with peaks around 7.00, 11.00 and 17.00 [41]. Clearly our model only holds if people are actually visiting patches when the mosquitoes are active. Asynchrony would effectively remove the associated mosquito and host subpopulations from the active transmission cycle. This adjustment would reduce the reproductive numbers, and bias transmission toward patches where behavior is synchronized, but the qualitative dynamics we have presented should remain unchanged. Note, however, that diurnal peaks in biting do not mean there is no biting at all outside of these times. Furthermore, laboratory experiments have found that, depending on diet, the biting behavior of *Ae. aegypti* may be opportunistic and not follow normal crepuscular or diurnal rhythms [42]. Field researchers have noted that *Ae. aegypti* is highly anthropophilic, and mosquito biting patterns tend to depend on human activity [43]. These observations suggest that if people only visit a patch outside of normal peak biting times mosquitoes will be either be absent or will have adjusted their biting behavior to synchronize.

We have assumed that mosquitoes do not move at all. Some gradual diffusion of the mosquito population might be expected. However, we feel that mosquito movement will only become important as we slide from urban to rural environments where human movement distances on short time scales may be closer to that of mosquitoes. Nevertheless, in the modern world, even remote villages are often connected to other villages by mass transportation. So on longer time scales, human movement is likely to dominate the epidemiological dynamics.

We also assumed that each mosquito population persists indefinitely at demographic equilibrium. This should be a reasonable approximation over intermediate time periods since we consider fairly large patch population sizes of the order 1000. It would, however, be interesting to modify our framework to consider mosquito populations that are transient due to micro-environment variability. From the pathogen perspective, such transience in the mosquito distribution would create a dynamic landscape for colonization. A number of dynamic landscape metapopulation models have been developed for ecological contexts. The general conclusion is that turnover in patch suitability for colonization has a detrimental impact on persistence of the metapopulation. The distribution of the refractory period, during which the patch cannot be re-colonized, is of key importance [44], [45], [46]. The potential impact of landscape dynamics in our model is difficult to assess. It is likely to depend on the timescale of the mosquito patch extinction and regeneration dynamics relative to the disease transmission dynamics. Very fast turnover of mosquito populations would severely limit their role as pathogen reservoirs. Slower turnover would complicate the dynamics but we suspect the main qualitative results we have described would still hold. Further work is required for confirmation.

Finally, we assumed all people continue to move at the same rate regardless of their infection status. We could relax this assumption to allow symptomatic people to quarantine themselves by returning to the home patch and remaining there until recovered. This modification would reduce both the number of people in a patch at any time and the proportion of these people that are infectious. However, the total number of infectious people is likely to be small compared with the total population size. If there was a major epidemic then radical changes in individual and governmental behavior would be anticipated anyway. Furthermore, at least in the case of dengue, the majority of infections are asymptomatic. For example, a prospective study of children in Bangkok, Thailand found that 87% of dengue positive cases were either asymptomatic or absent only one day from school [26].

Almost a century ago it was observed that people do not develop a disease where it is contracted or even close to that place [47]. Widespread rapid transit systems make that observation more relevant than ever. Metapopulation theory provides an excellent framework for understanding host-pathogen dynamics in structured environments. Here it has been used to show that the incidence and persistence of vector-borne diseases on relatively small spatial scales may be strongly influenced by infectious humans who remain mobile because the infection is mild or silent. Increased human movement on a local scale may be a key factor behind increased incidence of vector-borne diseases. The implication is that surveillance with the goal of controlling vector-borne disease may be a much greater challenge than originally anticipated. In modern cities daily travel is a way of life. Distant subpopulations of mosquitoes may be connected by this movement. A metapopulation is created that enhances a pathogen's resistance to eradication and complicates identification of the source of infection. Large, localized mosquito populations in areas that people visit regularly may be both reservoirs and hubs of infection, even if people only pass through those locations briefly. Increased human movement enhances the influence of such patches. So, ultimately successful public health intervention may need to focus on both hosts and vectors. Large mosquito populations that are also visited by a large fraction of the human population need to be identified. It is essential to employ surveillance strategies that reveal the variability in the distribution of mosquitoes and work to target areas where the mosquito population is significant and human movement is extensive. Costs may be reduced and efficiency improved if this surveillance is combined with a form of contact tracing for infected people. Mapping all their recent movements and comparing pathogen genotypes isolated from them and from mosquitoes may allow us to pinpoint the mosquito population from which they acquired the infection and others to which they may have transmitted it. Further study of networks formed by human movement in urban areas are called for, cell phone records are one potential source of such detailed information[48].

Here we have examined the hypothesis that people act as hosts and vectors of mosquito-borne diseases on a relatively small spatial scale. On regional, national and international scales the idea is already well accepted. For instance, Singapore receives imported dengue infections from high migration areas such as Malaysia, Indonesia and Thailand [3]. Hawaii gets several imports a year that sometimes lead to local transmission [12]. Dengue epidemics spread out from Bangkok as a traveling waves with a speed of 148 kilometers per month [49], a rate more easily reconciled with the movement of people than mosquitoes. The islands of the Caribbean may be thought of as patches connected by human travel. Dengue may persist there through a metapopulation process [50], [51]. Humans are by no means alone in acting as long distance vectors. Since its introduction in New York in 1999 West Nile virus has spread throughout North America. It may be an example of a pathogen transmitted by mosquitoes but disseminated by birds [52]. In most scientific fields a vector is a quantity that has a magnitude and direction. The term was first coined as a word to describe insects as intermediate agents in the transmission of disease in 1922 [1]. However, when thinking about pathogens transmitted when mosquitoes bite humans, maybe the roles are duplicated and, from the pathogen's point of view, it is also a case of man bites mosquito.

## Supporting Information

Supplementary Information S1 (0.58 MB PDF)
