# Supplementary material for: Man Bites Mosquito: Understanding the Contribution of Human Movement to Vector-Borne Disease Dynamics
Source: PLoS One. 2009 Aug 26;4(8):e6763. doi: 10.1371/journal.pone.0006763 (PMC2727792; doi:10.1371/journal.pone.0006763)
Supplement: Supplementary Information S1 — (0.58 MB PDF) [file pone.0006763.s001.pdf]

Supplementary Information

Distribution of mosquito population between patches

In order to introduce variation in the number of mosquitoes in each patch without changing the total number in the metapopulation we use the exponential distribution for x in the interval $[0, \xi]$, $F(x) = \lambda e^{-\lambda x}$, where λ controls the degree of skew. When λ is large the distribution is strongly skewed and the area under the curve on $[0, \xi]$ is very close to 1. However, when λ is close to 0, the distribution is more uniform and the area under the curve defined on $[0, \xi]$ may be much less than 1. Therefore we use the normalized function $G(x)$ constructed by dividing $F(x)$ by the total area under the curve on $[0, \xi]$:

$$G(x) = \frac{F(x)}{\int_0^{\xi} \lambda e^{-\lambda x} dx} = \frac{F(x)}{1 - e^{-\xi\lambda}} \qquad \text{S(1)}$$

Given n patches, the interval $[0, \xi]$ is divided into n equal subintervals by the partition $x_i = \xi(i-1)/n$, $i = 1 \ldots n+1$. The area under the curve $G(x)$ in each subinterval gives the proportion of the vector population associated with that patch and multiplying by the total vector population size gives the actual number of vectors in the patch:

$$N_i^v = \frac{\int_{x_i}^{x_{i+1}} \lambda e^{-\lambda x} dx}{\int_0^{\xi} \lambda e^{-\lambda x} dx} N_v = \frac{e^{-\lambda x_i} - e^{-\lambda x_{i+1}}}{1 - e^{-\xi\lambda}} N_v \qquad \text{S(2)}$$

Throughout this study $\xi = 120$.

Construction of equations for n patch model, no transit patch

The model integrates a SEIR framework for the host population and a SEI framework for the vector population [1] into a metapopulation structure in which distinct vector subpopulations are linked by host movement [2]. The total host population N^h is divided into n subpopulations N^h_j where $j = 1 \ldots n$ is the usual destination patch of that group. Each subpopulation is subdivided into a further $n + 1$ subpopulations N^h_{ij} where $i = 0 \ldots n$ is the current patch of those individuals. Hosts in patch 0 leave at rate ρ and travel to their usual destination patch with probability $(1 - \delta) + \delta/n$ and one of the other $n – 1$ patches each with probability δ/n. Hosts in patch $i \neq 0$ leave at rate τ and return directly to patch 0. Each host subpopulation is subdivided according to infection status: susceptible (S^h_{ij}), exposed (infected but not infectious, E^h_{ij}), infectious (I^h_{ij}) and recovered (R^h_{ij}). Hosts of all classes die at constant rate μ_h and are replaced with susceptible hosts. Infected hosts become infectious at rate ε_h. Infectious hosts recover at rate γ. Recovered hosts have complete lifelong immunity to re-infection. All hosts continue to move at the same rate regardless of their infection status. Each vector subpopulation is subdivided into susceptible (S^v_i), exposed (E^v_i) and infectious (I^v_i) classes. Vectors of all classes die at constant rate μ_v and are replaced with susceptible vectors. Exposed vectors become infectious at rate ε_v and remain in this class until they die. Vectors bite at rate β. So in patch i there are a total of βS^v_i bites by susceptible vectors. Considering the proportion of hosts in the patch that are infectious, the host-vector transmission rate is then:

$$\beta S_i^v \frac{\sum_{j=1}^{n} I_{ij}^h}{\sum_{k=1}^{n} N_{ik}^h} \qquad \text{S(3)}$$

Following the same reasoning, in patch i, the vector-host transmission rate for hosts with normal destination patch j is:

$$\beta I_i^v \frac{S_{ij}^h}{\sum_{k=1}^{n} N_{ik}^h} \qquad \text{S(4)}$$

The complete system of ordinary differential equation is thus:

$$
\begin{aligned}
&\dot{S}_{0j}^{h} = \mu_h (N_{0j}^{h} - S_{0j}^{h}) + \tau \sum_{i=1}^{n} S_{ij}^{h} - \rho S_{0j}^{h} \quad j = 1...n \\
&\dot{S}_{ij}^{h} = \mu_h (N_{ij}^{h} - S_{ij}^{h}) - \beta I_i^{v} \frac{S_{ij}^{h}}{\sum_{k=1}^{n} N_{ik}^{h}} - \tau S_{ij}^{h} + \frac{\delta}{n} \rho S_{0j}^{h} \quad i = 1...n, j = 1...n, i \neq j \\
&\dot{S}_{ii}^{h} = \mu_h (N_{ii}^{h} - S_{ii}^{h}) - \beta I_i^{v} \frac{S_{ii}^{h}}{\sum_{k=1}^{n} N_{ik}^{h}} - \tau S_{ii}^{h} + \frac{\delta}{n} \rho S_{0i}^{h} + (1-\delta) \rho S_{0i}^{h} \quad i = 1...n \\
&\dot{E}_{0j}^{h} = -(\varepsilon_h + \mu_h + \rho) E_{0j}^{h} + \tau \sum_{i=1}^{n} E_{ij}^{h} \quad j = 1...n \\
&\dot{E}_{ij}^{h} = \beta I_i^{v} \frac{S_{ij}^{h}}{\sum_{k=1}^{n} N_{ik}^{h}} - (\varepsilon_h + \mu_h + \tau) E_{ij}^{h} + \frac{\delta}{n} \rho E_{0j}^{h} \quad i = 1...n, j = 1...n, i \neq j \\
&\dot{E}_{ii} = \beta I_i^{v} \frac{S_{ii}^{h}}{\sum_{k=1}^{n} N_{ik}^{h}} - (\varepsilon_h + \mu_h + \tau) E_{ii}^{h} + \frac{\delta}{n} \rho E_{0i}^{h} + (1-\delta) \rho E_{0i}^{h} \quad i = 1...n \\
&\dot{I}_{0j}^{h} = \varepsilon_h E_{0j}^{h} - (\gamma + \mu_h + \rho) I_{0j}^{h} + \tau \sum_{i=1}^{n} I_{ij}^{h} \quad j = 1...n \\
&\dot{I}_{ij}^{h} = \varepsilon_h E_{ij}^{h} - (\gamma + \mu_h + \tau) I_{ij}^{h} + \frac{\delta}{n} \rho I_{0j}^{h} \quad i = 1...n, j = 1...n, i \neq j \\
&\dot{I}_{ii}^{h} = \varepsilon_h I_{ii}^{h} - (\gamma + \mu_h + \tau) I_{ii}^{h} + \frac{\delta}{n} \rho I_{0i}^{h} + (1-\delta) \rho I_{0i}^{h} \quad i = 1...n \\
&\dot{R}_{0j}^{h} = \gamma I_{0j}^{h} - (\mu_h + \rho) R_{0j}^{h} + \tau \sum_{i=1}^{n} R_{ij}^{h} \quad j = 1...n \\
&\dot{R}_{ij}^{h} = \gamma I_{ij}^{h} - (\mu_h + \tau) R_{ij}^{h} + \frac{\delta}{n} \rho R_{0j}^{h} \quad i = 1...n, j = 1...n, i \neq j \\
&\dot{R}_{ii}^{h} = \gamma I_{ii}^{h} - (\mu_h + \tau) R_{ii}^{h} + \frac{\delta}{n} \rho R_{0i}^{h} + (1-\delta) \rho R_{0i}^{h} \quad i = 1...n \\
&\dot{S}_i^{v} = \mu_v (N_i^{v} - S_i^{v}) - \beta S_i^{v} \frac{\sum_{j=1}^{n} I_{ij}^{h}}{\sum_{k=1}^{n} N_{ik}} \\
&\dot{E}_i^{v} = \beta S_i^{v} \frac{\sum_{j=1}^{n} I_{ij}^{h}}{\sum_{k=1}^{n} N_{ik}} - (\varepsilon_v + \mu_v) E_i^{v} \\
&\dot{I}_i^{v} = \varepsilon_v E_i^{v} - \mu_v I_i^{v}
\end{aligned}
\qquad \text{S(5)}
$$

Derivation of approximate system for *n* patches, no transit patch

To simplify the model we apply a method previously used for an epidemiological metapopulation model with direct transmission [2]. We assume that, as long as the time spent away from patch 0 is relatively short, the timescale of the travel dynamics is much faster than the timescale of the epidemiological dynamics. Therefore we approximate the population size of each host type (S^h_{ij}, E^h_{ij}, I^h_{ij}, R^h_{ij}) in each patch by assuming that they scale with the proportion of the total population expected to be in that patch at equilibrium. We then define S^h_j to be the total number of susceptible hosts with normal destination patch j, irrespective of their current location. E^h_j, I^h_j, R^h_j and N^h_j are defined similarly. So:

$$S_j^h = \sum_{i=0}^{n} S_{ij}^h,\ E_j^h = \sum_{i=0}^{n} E_{ij}^h,\ I_j^h = \sum_{i=0}^{n} I_{ij}^h,\ R_j^h = \sum_{i=0}^{n} R_{ij}^h, N_j^h = \sum_{i=0}^{n} N_{ij}^h \qquad \text{S(6)}$$

Note that the definitions for the vector population S^v_j, E^v_j, I^v_j are unchanged and relate to the number of mosquitoes actually present in patch j. Also, the sum for the total population size here is over all patches i where that host type is currently present and is not the same as the sum that appears in the denominator of the transmission terms of the original equations, which is over all destination patches j. Taking the derivatives of the new variable, all the terms involving τ and ρ cancel, leaving:

$$\begin{aligned}
\dot{S}_j^h &= \mu_h(N_j^h - S_j^h) - \beta \sum_{i=1}^{n} I_i^v \frac{S_{ij}^h}{\sum_{k=1}^{n} N_{ik}^h} \\
\dot{E}_j^h &= \beta \sum_{i=1}^{n} I_i^v \frac{S_{ij}^h}{\sum_{k=1}^{n} N_{ik}^h} - (\varepsilon_h + \mu_h)E_j^h \\
\dot{I}_j^h &= \varepsilon_h E_j^h - (\gamma + \mu_h)I_j^h \\
\dot{R}_j^h &= \gamma I_j^h - \mu_h R_j^h \\
\dot{S}_i^v &= \mu_v(N_i^v - S_i^v) - \beta S_i^v \frac{\sum_{j=1}^{n} I_{ij}^h}{\sum_{k=1}^{n} N_{ik}} \\
\dot{E}_i^v &= \beta S_i^v \frac{\sum_{j=1}^{n} I_{ij}^h}{\sum_{k=1}^{n} N_{ik}} - (\varepsilon_v + \mu_v)E_i^v \\
\dot{I}_i^v &= \varepsilon_v E_i^v - \mu_v I_i^v
\end{aligned} \qquad \text{S(7)}$$

We now wish to eliminate the *ij* groupings in the host population by writing them in terms of the *j* groupings. In order to estimate the proportion of the population in each

group we consider the disease free system, i.e. system (5) with $I^v{}_i = 0$ for all i. Then the susceptible population is equal to the entire population and the system is described by:

$$\begin{aligned}
\dot{S}^h_{0j} &= \tau\sum_{i=1}^{n} S^h_{ij} - \rho S^h_{0j} = \tau S^h_j - (\tau+\rho)S^h_{0j} \\
\dot{S}^h_{ij} &= -\tau S^h_{ij} + \frac{\delta}{n}\rho S^h_{0j} \quad i \neq j \\
\dot{S}^h_{ii} &= -\tau S^h_{ii} + \left(\frac{\delta}{n} + (1-\delta)\right)\rho S^h_{0j} = -\tau S^h_{ii} + \left(1 - \frac{(n-1)\delta}{n}\right)\rho S^h_{0j}
\end{aligned} \qquad \text{S(8)}$$

Setting the derivatives equal to 0 gives:

$$S^h_{0j} = \left(\frac{\tau}{\tau+\rho}\right)S^h_j, \quad S^h_{ij} = \left(\frac{\rho}{\tau+\rho}\right)\left(\frac{\delta}{n}\right)S^h_j, \quad S^h_{ii} = \left(\frac{\rho}{\tau+\rho}\right)\left(1 - \frac{(n-1)\delta}{n}\right)S^h_j \qquad \text{S(9)}$$

The I, E and R hosts groups simplify in the same way. Furthermore, we assume that the host travel dynamics are symmetric, so the number of hosts visiting each patch is the same for all patches: N/n. It follows that the total number of hosts of each group $\sum_{k=1}^{n} N^h_{ik}$ is the same for all patches and so $\sum_{k=1}^{n} N^h_{ik} = \left(\frac{\rho}{\tau+\rho}\right)\frac{N^h}{n}$.

Substituting all of these approximations back into the condensed system (7) gives the approximate system:

$$\begin{aligned}
\dot{S}^h_j &= \mu_h\left(\frac{N^h}{n} - S^h_j\right) - \beta\frac{S^h_j}{N^h}\left(\delta\sum_{i=1}^{n} I^v_i + n(1-\delta)I^v_j\right) \\
\dot{E}^h_j &= \beta\frac{S^h_j}{N^h}\left(\delta\sum_{i=1}^{n} I^v_i + n(1-\delta)I^v_j\right) - (\varepsilon_h + \mu_h)E_j \\
\dot{I}^h_j &= \varepsilon_h E^h_j - (\gamma + \mu_h)I^h_j \\
\dot{R}^h_j &= \gamma I^h_j - \mu_h R^h_j \\
\dot{S}^v_j &= \mu_v(N^v_j - S^v_j) - \frac{\beta S^v_j}{N^h}\left(\sum_{i=1,}^{n} \delta I^h_i + n(1-\delta)I^h_j\right) \\
\dot{E}^v_j &= \frac{\beta S^v_j}{N^h}\left(\sum_{i=1}^{n} \delta I^h_i + n(1-\delta)I^h_j\right) - (\varepsilon_v + \mu_v)E^v_j \\
\dot{I}^v_j &= \varepsilon_v E^v_j - \mu_v I^v_j
\end{aligned} \qquad \text{S(10)}$$

This simplifies more than the direct transmission model considered by [2]. In that model it is necessary to estimate the probability of two hosts meeting in a patch. Here the vector facilitates the meeting indirectly so we only need to estimate the probability of a host meeting a vector and the vectors never leave the patch.

Basic reproductive number for model with one destination patch

Using the next generation method [3,4], the global reproductive number of the model with one destination patch is

$$R_0^2 = \frac{\beta^2 N^v}{N^h}\left(\frac{\varepsilon_h}{\varepsilon_h+\mu_h}\right)\left(\frac{1}{\gamma+\mu_h}\right)\left(\frac{\varepsilon_v}{\varepsilon_v+\mu_v}\right)\left(\frac{1}{\mu_v}\right)\left(\frac{(\rho+\tau)(\mu_h^2+2\mu_h\rho+\rho^2+\varepsilon_h(\mu_h+\rho)+\gamma(\mu_h+\varepsilon_h+\rho)+\rho\tau)}{\rho(\gamma+\mu_h+\rho+\tau)(\varepsilon_h+\mu_h+\rho+\tau)}\right)$$

S(11)

The first five terms appear in the classical R_0 for a host-vector system and give the expected number of transmission events before recovery or death if the initial infection is in patch 1 and the host population does not move. Quarantine of symptomatic infections would scale this quantity. The interpretation of these terms has been discussed extensively elsewhere [1,3]. The final term is the most relevant to this study. It takes into account the expected proportion of the infectious period an infected host actually spends in patch 1 [5].

Basic reproductive numbers for model with three destination patches

The global reproductive number for the model with three destination patches can be calculated numerically using the next generation method. In addition, we define the host reproductive number $R_0{}^h{}_j$ as the number of secondary host infections resulting from a single infected host with normal destination *j*. The vector reproductive number $R_0{}^v{}_j$ is defined similarly. As set out in detail below:

$$R_{0j}^h = \frac{\beta^2}{N^h}\left(\frac{\varepsilon_h}{\varepsilon_h+\mu_h}\right)\left(\frac{1}{\gamma+\mu_h}\right)\left(\frac{\varepsilon_v}{\varepsilon_v+\mu_v}\right)\left(\frac{1}{\mu_v}\right)\left(n(1-\delta)N_j^v+\delta N^v\right) = \Lambda\left(n(1-\delta)N_j^v+\delta N^v\right) \qquad \text{S(12)}$$

$$R_{0j}^v = \frac{\beta^2}{N^h}\left(\frac{\varepsilon_h}{\varepsilon_h+\mu_h}\right)\left(\frac{1}{\gamma+\mu_h}\right)\left(\frac{\varepsilon_v}{\varepsilon_v+\mu_v}\right)\left(\frac{1}{\mu_v}\right)(n(1-\delta)^2N_j^v+(2-\delta)\delta N^v) = \Lambda(n(1-\delta)^2N_j^v+(2-\delta)\delta N^v) \qquad \text{S(13)}$$

As before, the terms compounded into Λ also appear in the classical R_0 for a host-vector system and here we focus instead on the terms involving δ. Beginning with $R_0{}^h{}_j$, suppose there is initially one infected host that normally travels to patch *j*. The expected number of secondary infections in hosts that normally travel to patch *j* is:

$$\Lambda\left[\left(n(1-\delta)^2+2\delta(1-\delta)\right)N_j^v+\frac{\delta^2}{n}N^v\right] \qquad \text{S(14)}$$

Here $n(1-\delta)^2+2\delta(1-\delta)N_j^v$ corresponds to a transmission cycle entirely within patch j. The initial type *j* host infects vectors in patch *j* that go on to infect hosts of type *j*. The term $(\delta^2/n)N^v$ corresponds to a transmission cycle that only involves type *j* hosts, but occurs entirely outside of patch *j*. The initial type *j* host travels to a random patch and infects the local vectors which then infect other type *j* hosts that travel to this patch. The sum of these terms represents the local maintenance of disease within the host population

associated with patch j. The expected number of secondary infections in hosts that normally travel to patches beyond j is:

$$\Lambda\left[(n-2)\delta(1-\delta)N_j^v + \left(\delta - \frac{\delta^2}{n}\right)N^v\right] \qquad \text{S(15)}$$

Here $(n - 2)\delta(1 - \delta)N_j^v$ corresponds to a transmission cycle in which patch j vectors spread the infection to other host types. The initial infected host infects vectors in patch j, which then infect hosts that are not of type j but are visiting patch j. The term $(\delta - \delta^2/n)N^v$ corresponds to a transmission cycle in which the type j host spreads the infections to other patches. The initial infected host travels to patches other than j and infects the local vectors which then infect hosts of type other than j visiting that patch. The sum of these terms represents the spread of disease to host populations associated with other patches.

Turning to $R_0{}^{\mathrm{v}}{}_{\mathrm{j}}$, suppose there is initially a single infected vector in patch j. Then the expected number of secondary vector infections within patch j is:

$$\Lambda\left[\left(n(1-\delta)^2 + 2\delta(1-\delta)\right)N_j^v + \delta^2 N_j^v\right] \qquad \text{S(16)}$$

Here $n(1 - \delta)^2 + 2\delta(1 - \delta)N_j^v$ corresponds to a transmission cycle entirely contained within patch j. The term δ^2/N_j^v corresponds to hosts of type other than j becoming infected while visiting patch j and either remaining in, or returning to, patch j to re-infect the local mosquito population. The sum of these terms represents the local maintenance of disease within the patch j vector population. The expected number of secondary vector infections in patches beyond j is:

$$\Lambda\left[2\delta\left(1-\frac{n-1}{n}\delta\right)\left(N^v - N_j^v\right) + \left(\frac{n-2}{n}\delta^2\right)\left(N^v - N_j^v\right)\right] \qquad \text{S(17)}$$

which simplifies to:

$$\Lambda\left[\delta(2-\delta)\left(N^v - N_j^v\right)\right] \qquad \text{S(18)}$$

In (17) the term $2\delta(1 - (n - 1)\delta/n)(N^v - N_j^v)$ corresponds to a transmission cycle in which type j hosts spread the infection when, after being infected in patch j, they travel to a different patch and infect the local vector population. The term $(n - 2)\delta^2/n)(N^v - N_j^v)$ corresponds to a transmission cycle in which hosts of type other than j spread the infection by visiting patch j, becoming infected, and then traveling to a different patch. The sum of these terms represents the spread of disease to vector populations in other patches.

Modifications for model with *n* destination patches and transit patch
In order to include a transit patch in the model we introduce the host variables $S^h{}_{Aj}$, $E^h{}_{Aj}$, $I^h{}_{Aj}$, $R^h{}_{Aj}$ and the vector variables $S^v{}_A$, $E^v{}_A$, $I^v{}_A$. Here $S^h{}_{Aj}$ is the number of susceptible hosts with usual destination patch j currently in the transit patch A, $S^v{}_A$ is the number of susceptible vectors in patch (A) and the other variables are defined in the obvious way. Additional differential equations for these transit patch variables are constructed in the same way as for the destination patches. All other equations are the same as for the original model except that all fluxes out of patch 0 are into patch A and at rate ρ_1 rather than ρ, all fluxes into patches $i = 1\ldots n$ are from patch A, rather than patch 0, and at rate ρ_2 rather than ρ. Thus the system with a transit patch is described by:

$$
\begin{aligned}
\dot{S}^h_{Aj} &= \mu_h(N^h_{Aj} - S^h_{Aj}) - \beta I^v_A \frac{S^h_{Aj}}{\sum_{k=1}^{n} N^h_{Ak}} - \rho_2 S^h_{Aj} + \rho_1 S^h_{0j} \\
\dot{E}^h_{Aj} &= \beta I^v_A \frac{S^h_{Aj}}{\sum_{k=1}^{n} N^h_{Ak}} - (\varepsilon_h + \mu_h + \rho_2)E^h_{Aj} + \rho_1 E^h_{0j} \\
\dot{I}^h_{Aj} &= \varepsilon_h E^h_{Aj} - (\gamma + \mu_h + \rho_2) I^h_{Aj} + \rho_1 I^h_{0j} \\
\dot{R}^h_{Aj} &= \gamma I^h_{Aj} - (\mu_h + \rho_2) R^h_{Aj} + \rho_1 R^h_{0j} \\
\dot{S}^v_A &= \mu_v(N^v_A - S^v_A) - \beta S^v_A \frac{\sum_{j=1}^{n} I^h_{Aj}}{\sum_{k=1}^{n} N^h_{Ak}} \\
\dot{E}^v_A &= \beta S^v_A \frac{\sum_{j=1}^{n} I^h_{Aj}}{\sum_{k=1}^{n} N^h_{Ak}} - (\varepsilon_v + \mu_v) E^v_A \\
\dot{I}^v_A &= \varepsilon_v E^v_A - \mu_v I^v_A
\end{aligned}
\qquad \text{S(19)}
$$

On the assumption that the host movement dynamics are fast compared to the infection and demographic dynamics we can approximate the variables with multiple subscripts in these equations in terms of the compound variables $S^h{}_i$, $E^h{}_i$, $I^h{}_i$, $R^h{}_i$ which represent hosts that normally travel to patch i regardless of their present location. Then the system reduces to:

$$
\begin{aligned}
\dot{S}_j^h &= \mu_h(\frac{N^h}{n} - S_j^h) - \beta\frac{S_j^h}{N^h}\left(\delta\sum_{i=1}^{n} I_i^v + n(1-\delta)I_j^v + I_A^v\right) \\
\dot{E}_j^h &= \beta\frac{S_j^h}{N^h}\left(\delta\sum_{i=1}^{n} I_i^v + n(1-\delta)I_j^v + I_A^v\right) - (\varepsilon_h + \mu_h)E_j^h \\
\dot{I}_j^h &= \varepsilon_h E_j^h - (\gamma + \mu_h)I_j^h \\
\dot{R}_j^h &= \gamma I_j^h - \mu_h R_j^h \\
\dot{S}_j^v &= \mu_v(N_j^v - S_j^v) - \frac{\beta S_j^v}{N^h}\left(\delta\sum_{i=1}^{n} I_i^h + n(1-\delta)I_j^h\right) \\
S_A^v &= \mu_v(N_A^v - S_A^v) - \frac{\beta S_A^v}{N^h}\sum_{i=1}^{n} I_i^h \\
\dot{E}_j^v &= \frac{\beta S_j^v}{N^h}\left(\delta\sum_{i=1}^{n} I_i^h + n(1-\delta)I_j^h\right) - (\varepsilon_v + \mu_v)E_j^v \\
\dot{E}_A^v &= \frac{\beta S_A^v}{N^h}\sum_{i=1}^{n} I_i^h - (\varepsilon_v + \mu_v)E_A^v \\
\dot{I}_j^v &= \varepsilon_v E_j^v - \mu_v I_j^v
\end{aligned}
\qquad \text{S(20)}
$$

Basic reproductive number for model with *n* identical patches plus transit patch

If we assume that the total vector population excluding the transit patch subpopulation is uniformly distributed between the *n* destination patches, so each patch contains N^v/n vectors, and hosts always travel to the same destination patch, so $\delta = 0$, then the basic reproductive number of the system found using the next generation method is:

$$
(R_0^T)^2 = \frac{\beta^2(N^v + N_A^v)\varepsilon_h\varepsilon_v}{N^h(\gamma + \mu_h)(\varepsilon_h + \mu_h)\mu_v(\varepsilon_v + \mu_v)} \qquad \text{S(21)}
$$

If $N^v_A = 0$ and vectors are absent from the transit patch, this reduces to the classical R_0 for a host-vector system with a host vector ratio of N^v/N^h.

References

1. Anderson RM, May RM (1991) Infectious diseases of humans : dynamics and control. Oxford ; New York: Oxford University Press. viii, 757 p. p.
2. Keeling MJ, Bjornstad ON, Grenfell BT (2004) Metapopulation dynamics of infectious diseases. In: Hanski I, Gaggiotti OE, editors. Ecology, genetics, and evolution of metapopulations. Burlington, MA: Elsevier. pp. xix, 696 p.
3. Diekmann O, Heesterbeek JAP (2000) Mathematical epidemiology of infectious diseases : model building, analysis, and interpretation. Chichester ; New York: John Wiley. xvi, 303 p. p.
4. van den Driessche P, Watmough J (2002) Reproduction numbers and sub-threshold endemic equilibria for compartmental models of disease transmission. Math Biosci 180: 29-48.
5. Gyllenberg M, Hanski I, Hastings A (1997) Structured metapopulation models. In: Hanski I, Gilpin ME, editors. Metapopulation biology : ecology, genetics, and evolution. San Diego, CA: Academic Press. pp. xvi, 512 p.
